# Adult Neural Stem Cell Migration Is Impaired in a Mouse Model of Alzheimer’s Disease

**DOI:** 10.1007/s12035-021-02620-6

**Published:** 2021-12-11

**Authors:** Daniel Esteve, María Micaela Molina-Navarro, Esther Giraldo, Noelia Martínez-Varea, Mari-Carmen Blanco-Gandia, Marta Rodríguez-Arias, José Manuel García-Verdugo, José Viña, Ana Lloret

**Affiliations:** 1 Freshage Research Group, Department of Physiology, Faculty of Medicine, University of Valencia, Valencia, Spain; 2 CIBERFES, Institute of Health Research-INCLIVA, Valencia, Spain; 3 Laboratory of Comparative Neurobiology, Instituto Cavanilles de Biodiversidad Y Biologia Evolutiva, University of Valencia, CIBERNED, Paterna, Valencia Spain; 4 Neuronal and Tissue Regeneration Laboratory, Centro de Investigación Príncipe Felipe, Valencia, Spain; 5 Departament of Biotecnology, Universitat Politècnica de València, Valencia, Spain; 6 Institute of Experimental Medicine, Czech Academy of Sciences, Prague, Czech Republic; 7 Second Faculty of Medicine, Charles University, Prague, Czech Republic; 8 Department of Psychobiology, Faculty of Psycology, University of Valencia, Valencia, Spain

**Keywords:** Subventricular zone, Beta-amyloid toxicity, Neurogenesis, Senescence, Olfaction

## Abstract

Neurogenesis in the adult brain takes place in two neurogenic niches: the ventricular-subventricular zone (V-SVZ) and the subgranular zone. After differentiation, neural precursor cells (neuroblasts) have to move to an adequate position, a process known as neuronal migration. Some studies show that in Alzheimer’s disease, the adult neurogenesis is impaired. Our main aim was to investigate some proteins involved both in the physiopathology of Alzheimer’s disease and in the neuronal migration process using the APP/PS1 Alzheimer’s mouse model. Progenitor migrating cells are accumulated in the V-SVZ of the APP/PS1 mice. Furthermore, we find an increase of Cdh1 levels and a decrease of Cdk5/p35 and cyclin B1, indicating that these cells have an alteration of the cell cycle, which triggers a senescence state. We find less cells in the rostral migratory stream and less mature neurons in the olfactory bulbs from APP/PS1 mice, leading to an impaired odour discriminatory ability compared with WT mice. Alzheimer’s disease mice present a deficit in cell migration from V-SVZ due to a senescent phenotype. Therefore, these results can contribute to a new approach of Alzheimer’s based on senolytic compounds or pro-neurogenic factors.

## Background

Two neurogenic regions are present in the postnatal anterior brain: the ventricular- subventricular area of the lateral ventricles (V-SVZ) and the subgranular layer of the dentate gyrus of the hippocampus [1]. In these regions, cell divisions continue to occur throughout life. Complex interactions between intrinsic molecular programmes and extrinsic determinants induce neural stem cells and neural progenitors to proliferate, differentiate and migrate [2–4].

The cellular organization of the V-SVZ is very well described both in mice and humans. In adult mice, four principal cell types have been described: the ependymal cells, the astrocyte-like type B cells, the type C cells and the type A cells. Ependymal cells (type E cells) line the ventricular cavity of the lumen of ventricles and display numerous cilia that are responsible for the flow of the cerebrospinal fluid (CSF). The astrocyte-like type B cells have been identified as stem cells with self-renewal property [5] and are characterized by the presence of intermediate filaments in their cytoplasm and by nestin-positive immunoreactivity [6]. Type C cells are large cells with invaginated nuclei and immature ultrastructural characteristics. They are considered as ‘transit amplifying progenitors’ giving rise rapidly to migratory or type A cells. These cells are responsible for the formation of the granular and periglomerular neurons in the olfactory bulb [7]. More recently, cellular migration has been also described to other regions such as the *striatum*, *corpus callosum and nucleus accumbens* [8–10].

However, the cell proliferative capacity is limited and can be modified both by physiological and pathological processes. When the cell loses this function, it enters in a senescent state in which its proliferative capacity is irreversibly abolished, but its vital activities are still taking place [11]. The first cause of senescence to be described was the shortening of telomeres. Other causes such as DNA mutations, loss of methylation of the CDKN2A gene associated with ageing, cytotoxic agents and oxidative stress can give rise to this cellular stage [11]. All these causes lead to an increase in the expression of proteins that stop the cell cycle and mainly provoke morphological changes, cell growth, flattening and lengthening, vacuolization, an increase in lysosomal content and upregulation of β-galactosidase enzyme (SAβGAL) [12, 13].

In the last century, life expectancy has doubled, and therefore a significant increase in people reaching advanced ages has occurred. This advantage has also brought a change in the profile of the most prevalent diseases, being neurodegenerative and in particular Alzheimer's disease (AD), one of the main ones. Conventional pharmacological therapies fail to stop or recover brain functions, so in recent years research in tissue regeneration has become very relevant, and especially neuronal regeneration. However, although much progress has been made in the study of neural precursors, there is still a long way to go in this regard [14].

In AD, the most common form of dementia, a decrease in proliferation and neurogenesis occurs. This deficit in neurogenesis may promote cognitive defects in AD [15]. This phenomenon has been described in neurospheres from mice models and AD patients [16, 17], and also in different transgenic mice of AD [18, 19]. Even some recent studies are describing a reduction in hippocampal neurogenesis in AD patients [20]. However, some authors doubt the existence of adult neurogenesis in the *dentate gyrus* [21].

Concerning V-SVZ neurogenesis, it has been poorly studied both in AD patients and in AD animal models. In this study, we want to clarify the proliferative and migration capacity of V-SVZ cells in a mice model of AD, the APP/PS1 transgenic mice. We have observed that type A clumps cells accumulate and type E cells decrease in APP/PS1 compared to wild-type mice. Furthermore, migrating type A cells decrease in the rostral migratory stream (RMS) and are also arranged in a spatially different way respect wild-type mice closely to the ventricle surface. Biochemical changes that could explain this phenotype include: an increase in Cdh1 levels and a decrease in Cdk5, p35 and cyclin B1, indicating a cell cycle dysfunction. The number of V-SVZ cells in the S phase decreased in the AD model compared to WT mice. Finally, we show that accumulated cells are in a senescent state. We conclude that migration from V-SVZ to the olfactory bulb is impaired in APP/PS1 mice potentially contributing to a defective neuronal degeneration in AD.

## Materials and Methods

### Animal Models

B6C3-Tg (APPswe, PSEN1dE9)85Dbo (The Jackson Laboratory, USA) transgenic mice and wild-type (WT) mice from the same colony and littermates were used in this study at 3–6 months of age, both male and female. Mice were housed in groups (2–6 mice per cage) and maintained in a 12:12-h light–dark cycle at 23 ± 1 °C and 60% relative humidity with access to food and water ad libitum. All procedures were performed in accordance to the protocols approved by ‘Comisión de Ética en la Investigación Experimental, Vicerectorado de Investigación’ of the University of Valencia (ref: A1510738650191, A1550947177828).

We also performed an experimental AD mice model by intraventricular infusion of beta-amyloid (Aβ) peptide or PBS as control. Six-month-old WT mice were anaesthetized at 4% isoflurane (ISO) (C3H2ClF5O) (IsoFlo; Zoetis, Spain S.L.) and maintained at 1.5 to 2% ISO with an oxygen flow of 0.5 L/min. Anaesthesia was kept using a ‘T de Eyre’ system, and the position was fixed with a stereotaxic support (SR-6R; Nasishige, Japan). Atropine sulphate (0.05 mg/kg) (A0257; Sigma-Aldrich, USA) and buprenorphine (0.1 mg/kg) (Dechra Veterinary Products SLU) were also administrated intraperitoneally to avoid any pain or bradycardia symptoms. Between 0.05 and 0.1 mL of 5% lidocaine (Braun, Germany) was injected into the incision site, and the skull was surgically exposed. Small burr holes were drilled in the skull 0.1 mm anterior to bregma and 0.4 mm lateral to the mid-sagittal line. A Hamilton syringe (65,458–01; Hamilton, USA) was inserted into the burr holes reaching 2.2 mm below the surface of the skull where 1 μL of 10 μM oligomeric Aβ [22] was injected at a rate of 0.1 μL/min to avoid hydrocephalus (*n* = 4). For control mice, the same procedure with 1 μL of PBS was performed (*n* = 4). After the procedure, the wounds were stitched, the area disinfected with povidone iodine (Desinpov; AGB, Spain) and oxytetracycline/polimixin (Terramicin; FarmaSierra, Spain), and the awaking was monitored. In all the subjects, the conditions after the surgery were normal. After 24 h, the mice were sacrificed [23–26], the V-SVZ disaggregated and fresh cells used for analysis of β -galactosidase activity by flow cytometry.

### Primary Culture of Neural Precursor Cells

Neural stem cells (NSCs) were obtained from V-SVZ and cultured to grow neurospheres. Briefly, 3-month-old WT (*n* = 6) and APP/PS1 (*n* = 6) mice were sacrificed by cervical dislocation, and the brains were quickly removed. The V-SVZ were dissected, and the tissue obtained was mechanically dissociated to obtain a single cell suspension in 1 mL DMEM/F12 medium containing 0.6% glucose, 2 mmol/L L-glutamine, 9.6 g/mL putrescine, 6.3 ng/mL progesterone, 5.2 ng/mL sodium selenite, 0.025 mg/mL insulin, 0.1 mg/mL transferrin and 2 g/mL heparin. Cells were seeded at a final density of 25 × 103 cells in uncoated culture dishes (Corning, NY) in the presence of 20 ng/mL epidermal growth factor and 20 ng/mL of fibroblast growth factor. After 3 to 4 days of culture, colonies of clonally derived neurospheres started to appear. All the experiments were performed at the third passage. Aβ peptide (10 μM) or vehicle was added to the culture medium and incubated for 24 h.

To measure proliferation rate, we used 5-ethynyl-2′-deoxyuridine (EdU, Thermo Fisher) labelling, which detects directly de novo DNA synthesis during phase S. Briefly, neurospheres were plated into chamber slides and allowed to settle for 30 min. Then, half of the culture medium was replaced with fresh culture medium containing EdU at a final concentration of 10 μM, and cells were incubated for 3 h. Following incubation cells were fixed with formaldehyde, and EdU was detected following manufacturer’s instructions.

### Western Blot Analysis

Dissected V-SVZ from 6-month-old APP/PS1 and WT mice were freeze-clamped in liquid nitrogen and stored at − 80 °C until processed. V-SVZ were homogenized in lysis buffer (Tris: 76.5 mM; pH: 6.8; SDS: 2%; glycerol: 10%, supplemented with sodium orthovanadate (2 mM) and protease inhibitor (Sigma-Aldrich) in a proportion of 1 mL lysis buffer/100 mg brain tissue) and by using mechanical shear with a Potter-Glass-Teflon homogenizer (Rw20 DZM Homogenizer, Janke & Kunkel), at 2000 rpm in ice.

We measured the protein concentration present in the tissue homogenates by the Lowry protein assay (Sigma-Aldrich). Twenty micrograms of proteins from each sample was loaded in an SDS–polyacrylamide gel. Proteins were separated by electrophoresis and electro-transfected into a nitrocellulose membrane. The nitrocellulose membranes were blocked with 5% low-fat milk or 5% BSA (w/v) in 1 × TBS-Tween for 1 h at room temperature (RT). The next primary antibodies were incubated overnight at 4 °C diluted in the appropriated blocking solution: cdh1 (DCS-266 NBP1-54,465, 1:1000, Novus Biologicals), cdk5 (2506, Cell Signalling Technology, 1:1000), cyclin B1 (4138S, Cell Signalling Technology, 1:1000), γH2X (9718, Cell Signalling Technology, 1:1000), p16 (MA1-16,664, ThermoFisher, 1:500), α-tubulin (sc-8035, Santa Cruz Biotechnology, 1:8000) and GAPDH (G9545, Sigma, 1:20,000). Finally, membranes were incubated for 1 h at RT with the corresponding secondary antibody: anti-mouse IgG H&L Chain Specific Peroxidase Conjugate (401,215, Calbiochem, 1:6000), anti-rabbit IgG HRP-linked (7074S, Cell Signalling, 1:3000) or mouse monoclonal [KT98] Anti-Rat IgG2b H&L (HRP) (ab106750, Abcam, 1:1000). Signal detection was performed using ‘Luminata Classico Western HRP Substrate’ (WBLUC0500, Millipore Corporation, Billerica, USA). Western blot images were obtained with a biomolecular imager (ImageQuant™ LAS 4000, GE Healthcare Bio-Sciences) and densitometry accomplished using Image Gauge 4.0 software.

### Flow Cytometry Assays

V-SVZ tissue from 6-month-old APP/PS1 and WT mice was disaggregated and incubated at 37 °C for 35 min with EBSS medium enriched with papain, L-cysteine and EDTA. After centrifugation for 5 min at 600 g, the pellet was resuspended in DMEM/F12. Cell cycle characterization was performed using PI/RNASE Solution (Immunostep) and following the manufacturer’s recommended protocol. We also determined the content of mitochondrial hydrogen peroxide by MitoPY1 (SML0734, Sigma-Aldrich) which is an aryl-boronate derivate that in the presence of H_2_O_2_ releases a highly fluorescent product [27]. Finally, in infused Aβ model, β-galactosidase activity was measured using FluoReporter lacZ Flow Cytometry Kit (F-1930, Molecular Probes).

### Immunohistochemistry Assays

For immunohistochemistry assays, 6-month-old APP/PS1 and WT mice were anaesthetized with a lethal dose of sodium pentobarbital (100 mg/kg 20%; Dolethal Vetoquinol Madrid, Spain) and transcardially perfused with heparinized saline (0.1%, pH 7) and 4% paraformaldehyde solution in PBS (0.1 M, pH 7.4). Brains were post-fixed 24 h in 4% paraformaldehyde in PBS at 4 °C followed by 2 days in PBS with 30% sucrose at 4 °C for cryoprotection. Serial coronal or sagittal Sects. (40 μm) were obtained by freezing microtome (Leica) and stored in PBS with 30% sucrose at − 20 °C until used.

Brain sections were washed in PBS containing 0.5% Triton X-100 solution (PBS-Tx), 3 × at RT. Sections were blocked for 1 h at RT with PBS-Tx containing 10% of goat serum (NGS). After that, the tissue was incubated with the different primary antibodies overnight at 4 °C in PBS-Tx with 10% NGS. The following primary antibodies were used: p35/25 (C64B10) Rabbit mAb (2680, Cell Signalling Technology, 1:200), beta Galactosidase Monoclonal Antibody (S.394.9, Thermo Fisher, 1:3200), DIABLO Monoclonal Antibody (SMAC 17 1–87, ThermoFisher, 1:200), Anti-Doublecortin antibody (ab18723, Abcam, 1:1000), GFAP Polyclonal Antibody (OPA1-06,100, ThermoFisher, 1:200), mouse anti-polysialic acid-NCAM (PSA-NCAM) monoclonal antibody (MAB5324, Chemicon, 1:400) and p-ATM (sc-47739, Santa Cruz Biotechnology, 1:500). Afterward, sections were rinsed 3 × in PBS-Tx and incubated for 1.5 h at RT in a solution of 10% NGS in PBS-Tx with the secondary antibody: Anti-rabbit IgG (H + L), F(ab′)2 Fragment Alexa Fluor® 488 Conjugate (4412, Cell Signalling Tech, 1:1000); Anti-mouse IgG (H + L), F(ab′)2 Fragment Alexa Fluor® 647 Conjugate (4410, Cell Signalling Tech, 1:1000); Goat anti-Mouse IgG (H + L), Superclonal™ Recombinant Secondary Antibody Alexa Fluor 488 (A28175, Invitrogen, 1:1000); Goat pAb to Rb IgG Alexa Fluor 647 (ab150079, Abcam, 1:1000). Finally, sections were washed in PBS-Tx and incubated with DAPI (H1200, Vector, Burlingame, USA) for nuclear staining, mounted onto 0.5% pigskin gelatine-coated slides, and covered with 24 × 60 mm cover slips (Knittel GLASS) adding a drop of fluorescence mounting medium (S3023, DAKO, Barcelona, Spain).

### Image Acquisition and Data Analysis

Immunohistochemistry preparations were visualized with a confocal laser-scanning microscope (Leica TCS SP2 scanning multiphoton and confocal unit with an inverted DM1RB microscope; Ar–He–Ne). For quantification, 6 images were taken from at least 3 slices per staining and per condition, which was then averaged for each animal. Images containing the area of interest were analysed with ImageJ software. To compare protein levels, we used area proportions of the specific antibody signal normalized to background fluorescence. Pearson’s coefficient in the images from the co-localization analysis was performed by the script developed by Moser et al. [28], and the percentage of co-localization was obtained by the plugin from ImageJ: co-localization threshold.

### Odour Habituation Test

Olfactory deficits were screened following the procedure of Wesson et al. [29] using the odor-habituation 201 test [30] in 6-month-old APP/PS1 and WT mice. Odours (*n* = 7; heptanone, isoamyl acetate, limonen, ethyl valerate, pentanol, propyl butyrate and nonane; Sigma-Aldrich, St. Louis, MO) were diluted 1 × 10^−3^ in mineral oil and applied to a cotton-applicator stick. To prevent contact of the liquid odor with the animal, the stick was enclosed in an odourless plastic tube. This control method of odor presentation permits the minimization of visual and somato-sensory influences. Animals were tested in their own home cage to minimize potential influences of stress or anxiety on the behavioural measures. Testing took place during the dark phase of the animals’ day. Odours were delivered for 4 successive trials (1 block), 20 s each, separated by 30-s inter-trial intervals, by inserting the odor stick into a port on the top of the animal’s home cage. The number of approaches, defined as snout-oriented sniffing within 1 cm of the odor presentation port, was registered. Odor presentation and mice were tested in a counter-balanced order. For analysis of olfactory behaviour data, odor investigation durations within individual trials were collapsed across all odours. To calculate habituation index, the normalized investigatory values from all 4^th^ trial odor presentations were subtracted from the corresponding 1^st^ trial odor presentations.

### Electron Microscopy Analysis

For electron microscopy studies, 6-month-old mice were perfused with 2% paraformaldehyde-2.5% glutaraldehyde. We used 4 WT and 6 APP/PS1 mice, and 2 different brain levels of each mouse were studied. Brains were dissected out and post-fixed in the same fix solution. Coronal 200- μm sections were obtained using a vibratome (Leica VT-1000, Heidelberg, Germany) and post-fixed with 2% osmium, rinsed, dehydrated and embedded in Durcupan resin (Sigma, St Louis, MO, USA). Semithin Sects. (1.5 µm) were obtained, using an ultramicrotome (Leica EM UC-6, Heidelberg, Germany) with a diamond knife, and stained lightly with 1% toluidine blue to select the regions of interest. Ultra-thin Sects. (0.08 µm) were obtained using an ultramicrotome (Leica EM UC-6) with a diamond knife and stained with lead citrate (Reynolds solution). Images were analysed with a transmission electron microscope (FEI Tecnai G2 Spirit BioTwin, Hillsboro, OR, USA) using a digital camera (Morada, Soft Imaging System, Olympus, Tokyo, Japan).

### Statistical Analysis

Results are expressed as mean values ± SD, of at least three independent experiments. Generally, experiments were performed as duplicates, within each independent experiment. We used the Student’s *t* test to compare two means in parametric samples and the Mann–Whitney test for non-parametric samples. When we compared more than two means, we used the analysis of variance (ANOVA) test for parametric samples and the Kruskal–Wallis test for non-parametric samples. The null hypothesis was accepted at the level of *p* ≤ 0.05.

## Results

### Proliferation Rate Decreases in Neurospheres from APP/PS1 Mice

**Fig. 1:**
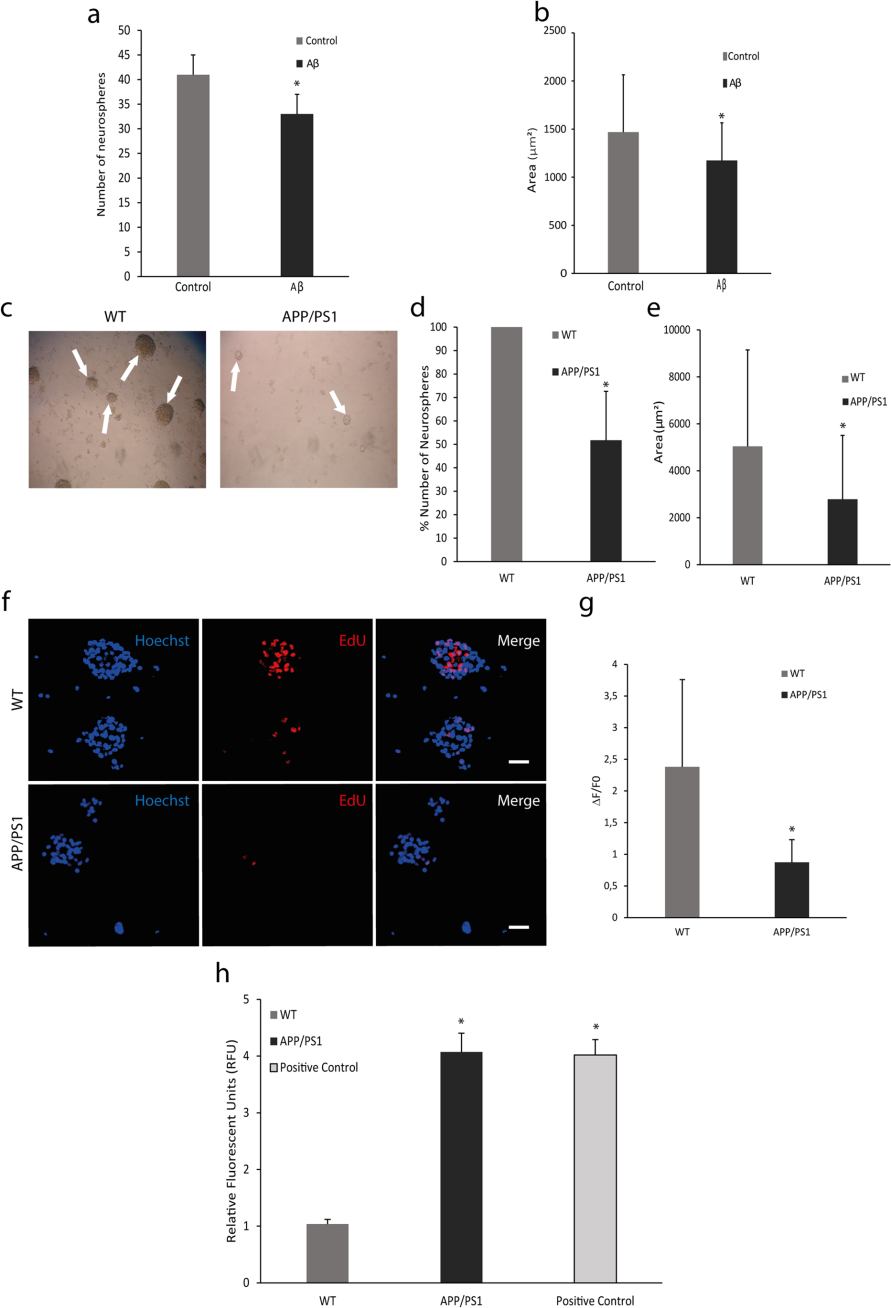
Proliferation rate and oxidative stress analysis. (**a)** Number of neurospheres from V-SVZ are less in the group incubated with 10 μM Aβ respect to the control group. Mean values ± SD. Student’s *t* test. *p = 0.0001 vs. control (*t* = 5.527, *df* = 12). *n* = 6. (**b)** Area (μm^2^) of neurospheres from WT mice shows a decrease after incubation with 10 μM Aβ. Mean values ± SD. Student’s *t* test. *p = 0.0163 vs. control (*t* = 2.459, *df* = 72). *n* = 6. (**c)** Representative images of neurospheres from WT and APP/PS1 mice. **(d)** Number of neurospheres from V-SVZ WT is higher than those from APP/PS1 mice. Mean values ± SD. Student’s *t* test. *p = 0.0003 vs. WT. (*t* = 5.974, *df* = 8). *n* = 6. (**e)** Area (μm^2^) measured in neurospheres decrease in APP/PS1 mice respect to WT. Mean values ± SD. Student’s *t* test. *p = 1 × 10^−11^ vs. WT (*t* = 6.895, *df* = 448). *n* = 6. (**f)** Representative images of neurospheres from WT and APP/PS1 mice labelled with EdU in red, which is incorporated to DNA during mitosis, and with Hoechst in blue marking nuclei. Scale bar = 50 μm. (**g)** Quantification of red fluorescence shows a decreased proliferation rate in neurospheres from APP/PS1 respect to WT. Mean values ± SD. Student’s *t* test. * *p* = 0.0098 vs. WT (*t* = 3.067, *df* = 12). *n* = 6. **(h)** Mitochondrial hydrogen peroxide (H_2_O_2_) production in SVZ cells is increased in APP/PS1 mice compared with WT. Mean values ± SD.ANOVA test. * *p* = 1 × 10^−6^ vs. WT (*F*_2,10_ = 361.3). *n* = 3

First, we wanted to find out if the Aβ peptide influences the proliferative capacity of neuronal stem cells in culture. For this purpose, we incubated neurospheres from V-SVZ of WT mice with 10 μM oligomeric Aβ for 24 h. Figure 1a shows that the number of neurospheres decreased after Aβ treatment. Moreover, the area of the neurospheres was lower in the Aβ-treated group compared to the PBS group (control) (Fig. 1b). These results were also verified in neurospheres from APP/PS1 mice (Fig. 1c–e). The incorporation of EdU to the DNA (Fig. 1f) showed a decrease in the proliferative capacity of neurospheres in APP/PS1 vs. WT (Fig. 1g). Then, we wanted to know if this decrease is due to the presence of reactive oxygen species (ROS)/oxidative stress. To determine in vivo oxidative stress in the V-SVZ cells from transgenic AD mice, we used a flow cytometer assay labelling with MitoPY1, a fluorescent dye that specifically determines H_2_O_2_ levels. We observed an increase of H_2_O_2_ levels in the transgenic model compared to controls (Fig. 1h).

### Migrating Precursor Cells Accumulate in V-SVZ from APP/PS1 Mice

**Fig. 2:**
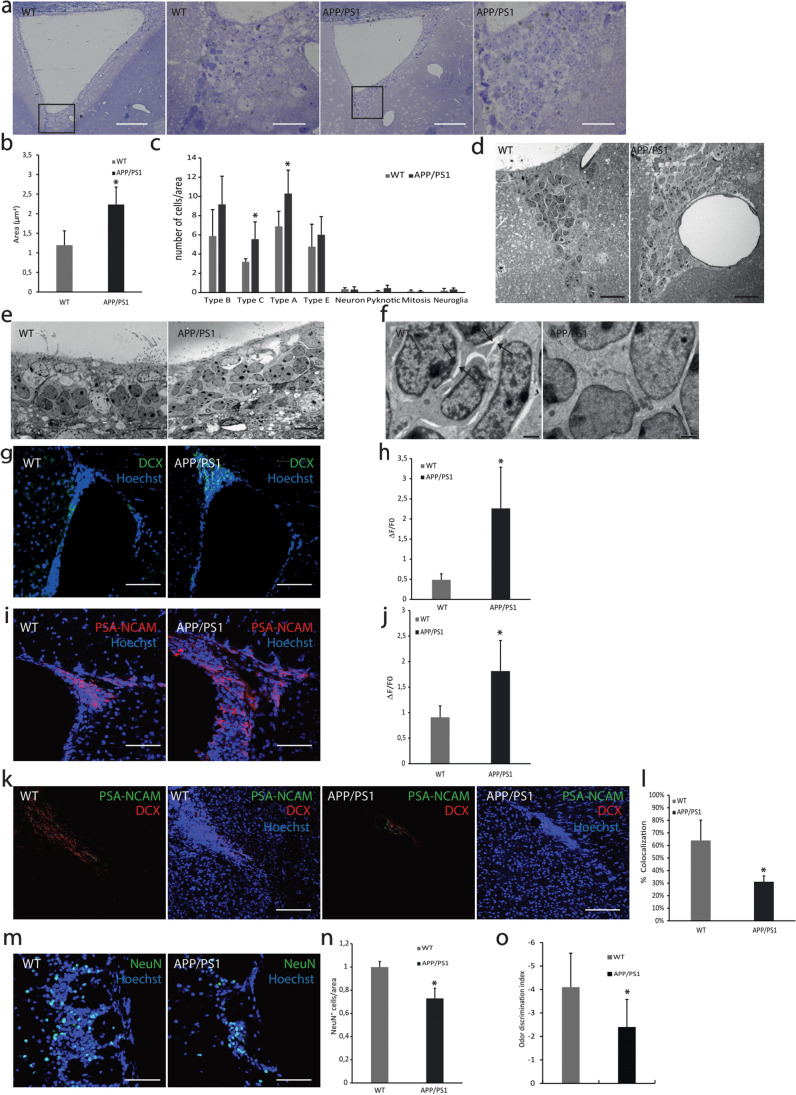
Morphological study of neural migration-related areas in APP/PS1 mice.** (a)** Images of toluidine blue-stained of V-SVZ slices show an accumulation of cells in APP/PS1 mice. Scale bar = 100 μm (1 and 3) 25 μm (2 and 4). *n* = 5. (**b)** Area (μm^2^) of the V-SVZ is increased in APP/PS1 mice due to the accumulation of cells. Scale bar = 100 μm. Mean values ± SD. Student’s *t* test. *p = 0.0031 vs. WT (*t* = 4.428, *df* = 7). *n* = 5. (**c)** Quantification of each cell type expressed respect to total cells and analysed area in V-SVZ by electron microscopy. Mean values ± SD. Student’s *t* test. *p = 0.0129 (*t* = 3.022, *df* = 10) (Type A vs. WT), *p = 0.0106 (*t* = 3.136, *df* = 10) (Type C vs. WT). *n* = 4. **(d)** Electron microscopy analysis of the V-SVZ reveals larger blood vessels in APP/PS1 than in WT mice. Scale bar = 20 μm. *n* = 5. **(e)** Electron microscopy analysis of the V-SVZ shows that type A cells are more disorganized in APP/PS1 compared to WT. Scale bar = 10 μm. *n* = 5. (**f)** Electron microscopy analysis of the V-SVZ displays spaces in the extracellular matrix in WT which are absent in APP/PS1. Scale bar = 1 μm. *n* = 5. (**g)** Confocal microscopy analysis of V-SVZ slices from WT and APP/PS1 mice labelled with anti-DCX (green) and with Hoechst (nuclei, blue). Representative images of three independent experiments. Scale bar = 75 μm. (**h)** Quantification of green fluorescence fold change shows an increase of DCX^+^ cells in APP/PS1 mice. Mean values ± SD. Student’s *t* test. *p = 0.0057 vs WT (*t* = 4.194, *df* = 6). *n* = 3. (**i)**. Representative images of PSA-NCAM (red) in V-SVZ from WT and APP/PS1 mice. Hoechst (blue) was used to identify cell nuclei. Scale bar = 75 μm. (**j)** Quantification of red fluorescence fold change shows an increase in PSA-NCAM expression in APP/PS1 mice. Data are shown as mean ± SD. Student’s *t* test. *p = 0.01 vs. WT (*t* = 3.500, *df* = 7). *n* = 4. (**k)** Representative images of the RMS from WT and APP/PS1 mice labelled with DCX (red) and PSA-NCAM (green). Hoechst (blue) was used to identify cell nuclei. Scale bar = 150 μm. (**l)** Quantification of co-localization of red (PSA-NCAM) and green (DCX) fluorescence shows a decrease of migratory cells in the RMS from APP/PS1 mice. Data are shown as mean ± SD. Student’s *t* test. *p = 0.0026 vs WT (*t* = 4.924, *df* = 6). *n* = 3. (**m)** Representative images of OB from WT and APP/PS1 mice labelled with NeuN (red). Hoechst (blue) was used to identify cell nuclei. Scale bar = 75 μm. (**n)** Quantification NeuN^+^ cells shows a decrease of mature neurons in the OB from APP/PS1 mice. Data are shown as mean ± SD. Student’s *t* test. *p = 0.0006 vs WT (*t* = 6.555, *df* = 6). *n* = 3. (**o)** Analysis of olfactory capacity shows a decrease in the APP/PS1 mice. Data are shown as mean ± SD. ANOVA test. *p = 0.012 vs WT (*F*_*1,1 8*_ = 7.811). *n* = 10

We wondered whether the reduced proliferation rate and oxidative stress induced by Aβ could affect the cellular organization of V-SVZ. For this purpose, we studied the structure of the V-SVZ of both WT and APP/PS1 mice. In APP/PS1 mice, we found cellular accumulation specifically in the dorsal part and in the fusion zone of the lateral ventricles (Fig. 2a). The total area of the V-SVZ was significantly increased in APP/PS1 mice compared to WT (Fig. 2b). To characterize these cellular clumps, we studied the ultrastructure of the regions (Fig. 2d–f). Based on the V-SVZ cellular classification reported by Doetsch et al., we obtained the percentage of each cellular type [6]. We observed a statistically significant increased number of type A cells, usually surrounded by big blood vessels, and an increase of type C cells in APP/PS1 compared to WT mice (Fig. 2c, d). Besides, APP/PS1 type A cells appear more disorganized than WT cells (Fig. 2e), and without extracellular matrix spaces needed for cell migration (Fig. 2f).

Immunolabeling confirmed that accumulated cells in APP/PS1 mice were type A cells since they expressed immaturity marker doublecortin (DCX) and the migrating neural progenitor cell marker, PSA-NCAM (Fig. 2g–j). Furthermore, to study the migration capacity of type A cells, we also analysed the content of DCX^+^/PSA-NCAM^+^ cells present at the proximal RMS. We observed a reduction of 32.9% in type A cells in the RMS from APP/PS1 mice compared with WT (Fig. 2k, l). Additionally, we measured the number of mature neurons (NeuN^+^) in the olfactory bulbs and observed a significant decrease in the AD transgenic mouse model (Fig. 2m, n). Importantly, APP/PS1 mice exhibited serious deficiency discriminating odours compared with WT animals at the same age (Fig. 2o).

### Cell Cycle Modifications in V-SVZ Cells from APP/PS1

**Fig. 3:**
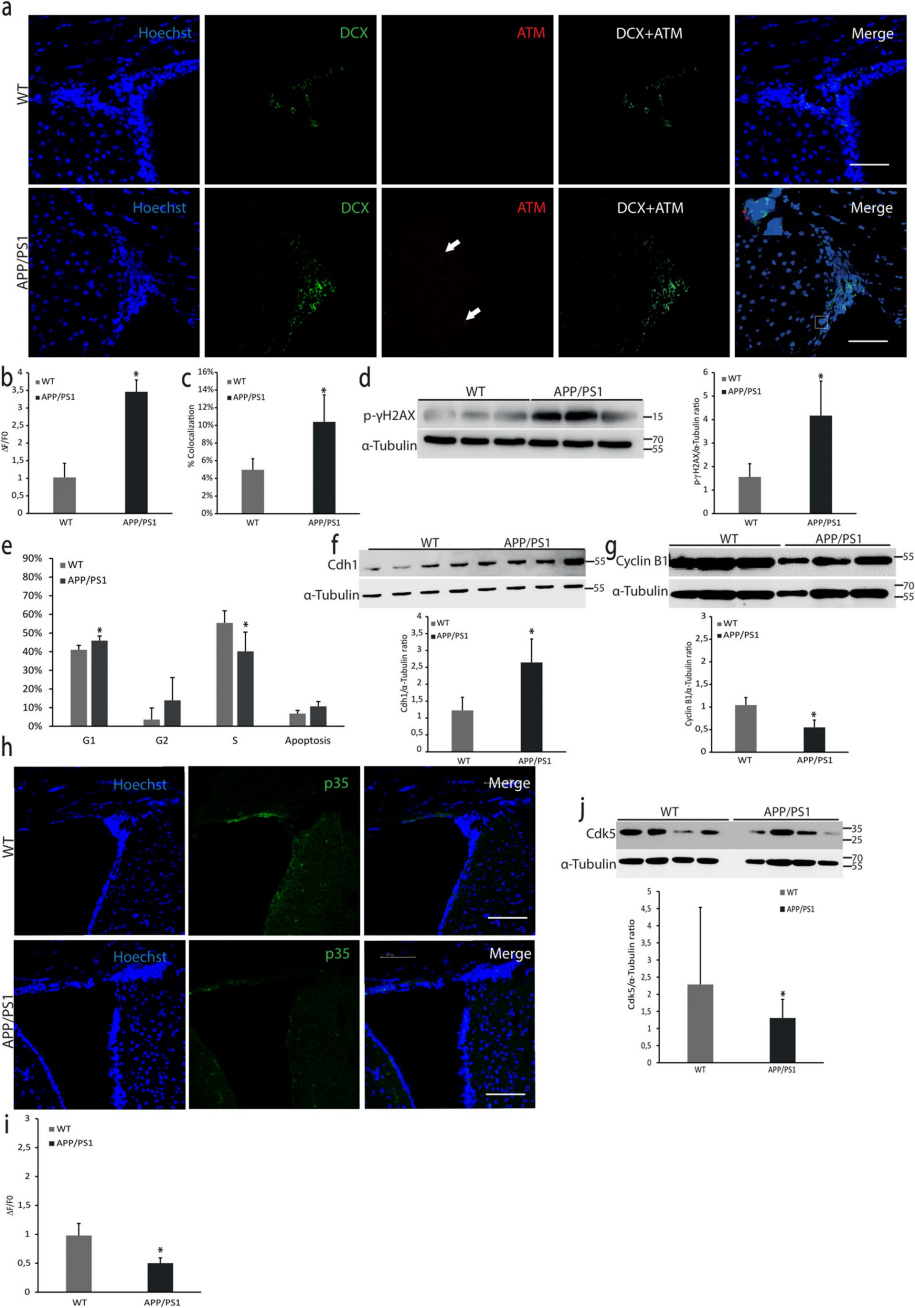
DNA damage and cell cycle study.** (a)** Confocal microscopy analysis of V-SVZ slices from WT and APP/PS1 mice. Anti-ATM in red, anti-DCX in green and nuclei stained with Hoechst in blue. Scale bar = 75 μm. (**b)** Quantification of red fluorescence fold change shows an increase of ATM content in APP/PS1 mice. Mean values ± SD. Student’s *t* test. *p = 0.0001 vs WT (*t* = 11.35, *df* = 6). *n* = 3. (**c)** Quantification of co-localization of ATM^+^ cells (red) and DCX^+^ cells (green) indicates that DCX^+^ cells express more ATM in APP/PS1 mice. Mean values ± SD. Student’s *t* test. *p = 0.007 vs WT (*t* = 4.017, *df* = 6). *n* = 3. (**d)** Western blot analysis of p-γH2AX levels in V-SVZ homogenates from WT and APP/PS1 mice. Blots of three independent experiments were quantified by densitometry and normalized with α-tubulin levels. Mean values ± SD are shown. Student’s *t* test. *p = 0.0066 vs. WT (*t* = 4.063, *df* = 6). **(e)** Flow cytometry characterization of the cell cycle of V-SVZ cells from WT and APP/PS1 mice shows that cells in the S phase are less in APP/PS1 than in WT mice. Mean values ± SD. Student’s *t* test. *p = 0.0224 vs WT (*t* = 3.054, *df* = 6). *n* = 3. (**f)** Representative images of western blot of Cdh1 in V-SVZ from WT and APP/PS1 mice. Blots were quantified by densitometry and normalized with α-tubulin levels; mean values ± SD of four independent experiments are shown. Student’s *t* test. *p = 0.0018 vs. WT (*t* = 4.595, *df* = 8). (**g)** Western blot of cyclin B1 in V-SVZ from WT and APP/PS1 mice. Blots were quantified by densitometry and normalized with α-tubulin levels. Mean values ± SD of three independent experiments are shown. Student’s *t* test. *p = 0.0021 vs. WT (*t* = 5.151, *df* = 6). (**h)** Representative images of p35 levels (green) in V-SVZ from WT and APP/PS1 mice. Hoechst (blue) was used to identify cell nuclei. Scale bar = 75 μm. (**i)** Quantification of green fluorescence fold change shows a decrease in the p35 expression in APP/PS1 mice. Mean values ± SD. Student’s *t* test. *p = 0.0021 vs WT (*t* = 5.133, *df* = 6). *n* = 3. (**j)** Western blot of Cdk5 levels shows a decrease in V-SVZ from APP/PS1 compared to WT mice. Data are shown as mean values ± SD. Student’s *t* test. *p = 0.0266 vs WT (*t* = 2.279, *df* = 54). *n* = 4

Taking together oxidative stress and cell accumulation in the V-SVZ of the transgenic model, we want to figure out possible molecular explanations. One of the main oxidative damage targets is DNA inducing possible alterations in the cell cycle. To determine DNA damage in V-SVZ tissue we measured the levels of p-ATM and its target, the phosphorylated histone γH2AX (p-γH2AX). Figure 3a–d shows an increase in p-ATM and p-γH2AX levels in APP/PS1 V-SVZ compared to WT. It is interesting to note that the increase in p-ATM co-localizes with DCX^+^ cells (Fig. 3a).

Increased levels of p-γH2AX are an established marker of replicative stress; therefore, we characterized the cell cycle by flow cytometry and some cell cycle-related proteins. We found a statistically significant increase of cells in the G1 phase (Fig. 3e) and a reduction of cells in the S phase in APP/PS1 mice compared with controls. Levels of Cdh1, an important co-factor of the cell cycle master regulator APC/C, increased in V-SVZ from APP/PS1 (Fig. 3f). APC/C is an E3 ubiquitin-ligase that labels proteins for degradation. This result fits with a cell cycle arrest in the G1 phase since APC/C-Cdh1 activity is crucial to passing through the G1 phase into the S phase. Consistent with this result, one of the APC/C-Cdh1 targets, cyclin B1 was significantly decreased in these animals (Fig. 3g). The Cdk5/p35 complex is a kinase that phosphorylates and therefore inactivates Cdh1. In this line, we observed a decrease in p35, the coactivator of Cdk5, (Fig. 3h–i), and in Cdk5 levels (Fig. 3j), indicating a reduction of Cdk5 activity.

### Study of Senescence and Apoptosis in V-SVZ

**Fig. 4:**
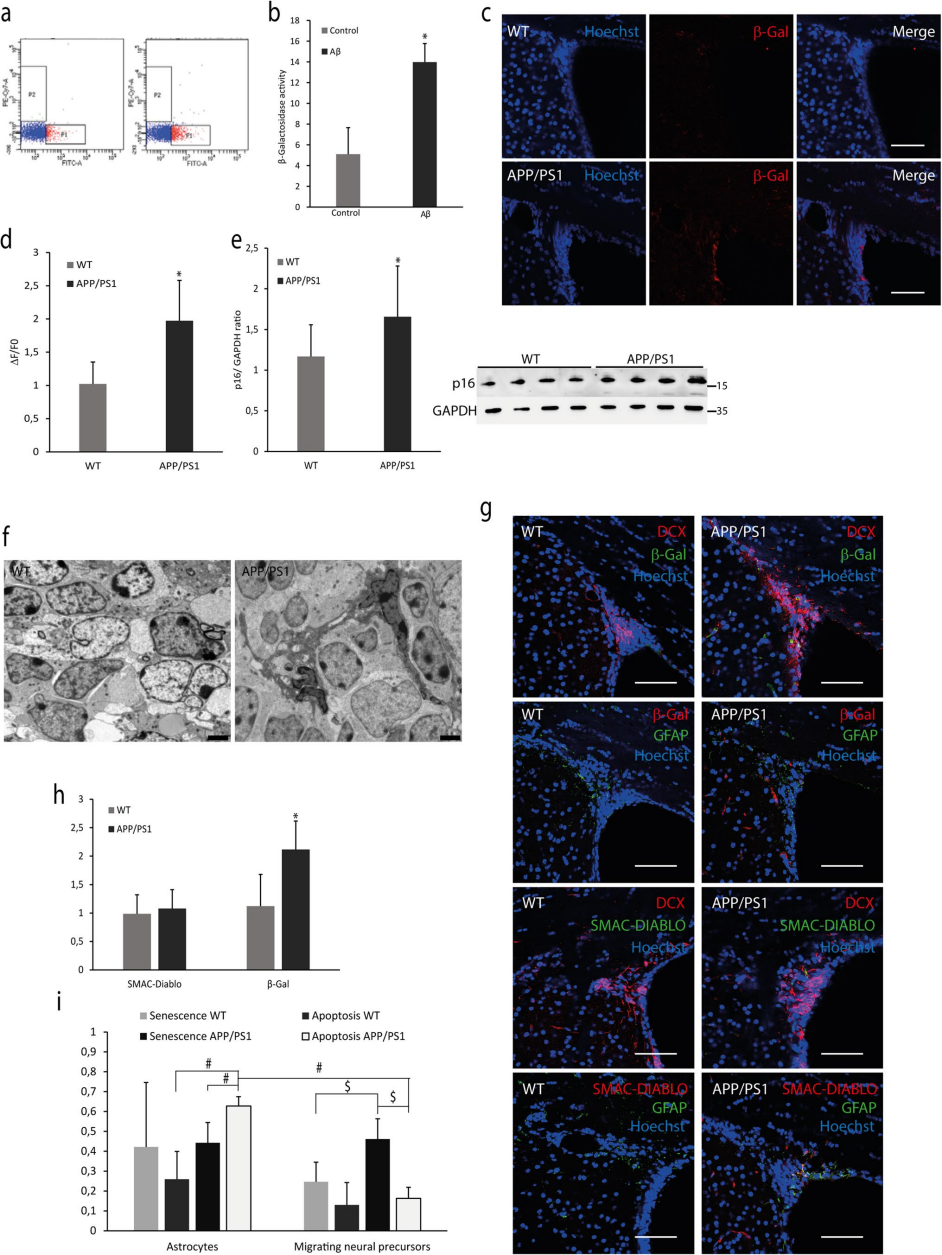
Senescence and apoptosis in the V-SVZ of APP/PS1.** (a)** Flow cytometry representative plots of β-Gal activity in V-SVZ cells measured after intraventricular infusion of Aβ. (**b)** Quantification of β-Gal activity reveals an increase in Aβ-treated mice compared to controls. Mean values ± SD. Student’s *t* test. *p = 0.0001 vs. control (*t* = 7.340, *df* = 8) *n* = 4. (**c)** Confocal microscopy representative images of β-Gal levels (red) in V-SVZ from WT and APP/PS1 mice. Hoechst (blue) was used to identify cell nuclei. Scale bar = 75 μm. (**d)** Quantification of red fluorescence fold change shows that β-Gal expression increases in V-SVZ APP/PS1 compared with WT. Mean values ± SD. Student’s *t* test. *p = 0.0044 vs. WT (*t* = 3.777, *df* = 9). *n* = 5. (**e)** Representative western blot image of p16 levels in V-SVZ from WT and APP/PS1 mice. Densitometry of bands shows an increase in p16 expression in APP/PS1 compared with WT. Data are shown as mean ± SD. Student’s *t* test. * *p* = 0.0231 vs. WT (*t* = 2.433, *df* = 23). *n* = 4. **(f)** Representative electron microscopy images of the V-SVZ from WT and APP/PS1. Magnification reveals the presence of apoptotic astrocytes in the V-SVZ from APP/PS1, and arrows indicate live astrocytes in WT mice. Scale bar = 2 µm. *n* = 5. (**g)** Representative images of β-Gal, DCX, GFAP and SMAC-DIABLO levels in V-SVZ from WT and APP/PS1 mice. Hoechst (blue) was used to identify cell nuclei. Scale bar = 75 μm. (**h)** Quantification of fluorescence fold change shows different levels of senescence (β-Gal) in the V-SVZ of APP/PS1 mice compared to WT. Mean values ± SD. Student’s *t* test. * *p* = 0.0055 vs. WT (*t* = 4.235, *df* = 6). *n* = 3. **(i)** Co-localization of apoptosis/senescence markers with astrocytes/migrating neural precursors markers. Data are shown as mean ± SD. ANOVA test. Statistics values are: *F*_*7,24*_ = 8.107; $ *p* = 0.0007 (apoptosis vs. senescence in APP/PS1 migrating neural precursors); *p* = 0.009 (migrating neural precursors senescence in APP/PS1 vs. WT). # *p* = 0.0069 (senescence vs. apoptosis in astrocytes from APP/PS1); *p* = 0.0001 (apoptosis in APP/PS1 migrating neural precursors vs. apoptosis in APP/PS1 astrocytes); *p* = 0.0009 (astrocytic apoptosis in APP/PS1 vs. WT). *n* = 3

To figure out whether the cell cycle arrest that we observe in the V-SVZ from APP/PS1 triggers a senescent state, we measured the amount of SAβGAL, one of the main markers of a senescent state. Microinjection of 10 µM soluble Aβ peptide in the lateral ventricles produces a SAβGAL phenotype in the V-SVZ of WT mice (Fig. 4a, b). We also measured this senescent state by immunofluorescence in V-SVZ tissue from WT and APP/PS1 mice, and we observed an increase in the last ones (Fig. 4c, d). Furthermore, we determined the content of p16, another senescence marker, in V-SVZ from WT and APP/PS1 mice, and we reported an increase in the transgenic model (Fig. 4e).

Finally, we observed by electron microscopy a relevant amount of apoptotic astrocytes in the V-SVZ from APP/PS1 (Fig. 4f). We decided to co-localize DCX (a neuronal immature marker) or GPAP (astrocyte marker) with a senescence marker (β-gal) or an apoptosis marker (Smac/DIABLO) (Fig. 4g–i). Our results showed that there are more senescent migrating neurons than apoptotic migrating neurons in the V-SVZ from APP/PS1 while astrocytes displayed more apoptosis than senescence. Furthermore, there is an increase of both DCX^+^/β-gal^+^ and GFAP^+^/DIABLO^+^ cells in APP/PS1 respect to WT (Fig. 4i). Therefore, we can assume that increase in the total senescence shown in the V-SVZ of APP/PS1 (Fig. 4d, h) comes from the accumulated type A cells.

## Discussion

Here, we show that Aβ toxicity induces NPCs to enter irreversible proliferative arrest with features of cellular senescence. APP/PS1 mice, which generate large amounts of brain Aβ peptide, have increased activation of ATM, an apical PI3K-like serine/threonine kinase that marks DNA lesions through phosphorylation of the histone H2A variant H2AX (γH2AX) [31–34]. DNA damage could be probably caused by the broadly demonstrated capacity of Aβ of increasing ROS production [35]. γH2AX is a marker of replicative senescence, cellular response to damaging stress characterized by a non-proliferative but viable state [36–38]. In our study we have observed that direct microinjection of Aβ inside the lateral ventricles increases the β-gal activity in V-SVZ cells, an established marker of senescence. Moreover, we show that V-SVZ type A cells from APP/PS1 mice accumulated in huge cellular clumps. These cells display an increase in SA-β-gal phenotype and p16 expression, indicating a senescence state. Type A cells are migratory cells (neuroblasts) that should be moved to the olfactory bulb along the RMS [1, 39]. APP/PS1 mice have a reduced number of type A cells in the RMS compared to WT. This result suggests a reduction in the olfactory bulb neurogenesis which fits with Scopa et al., which also reported an increase in neuroblast number in V-SVZ due to the presence of Aβ [40]. Moreover, we observe by electron microscopy that these senescent and accumulated cells are surrounded by a significant number of thick blood vessels. In this sense, angiogenesis has been linked with the senescent-associated secretome in many types of cells [41–43]. Furthermore, angiogenesis is related to the leading process of the migrating NPCs, suggesting that this interaction provides directional guidance to the NPCs [44].

**Fig. 5:**
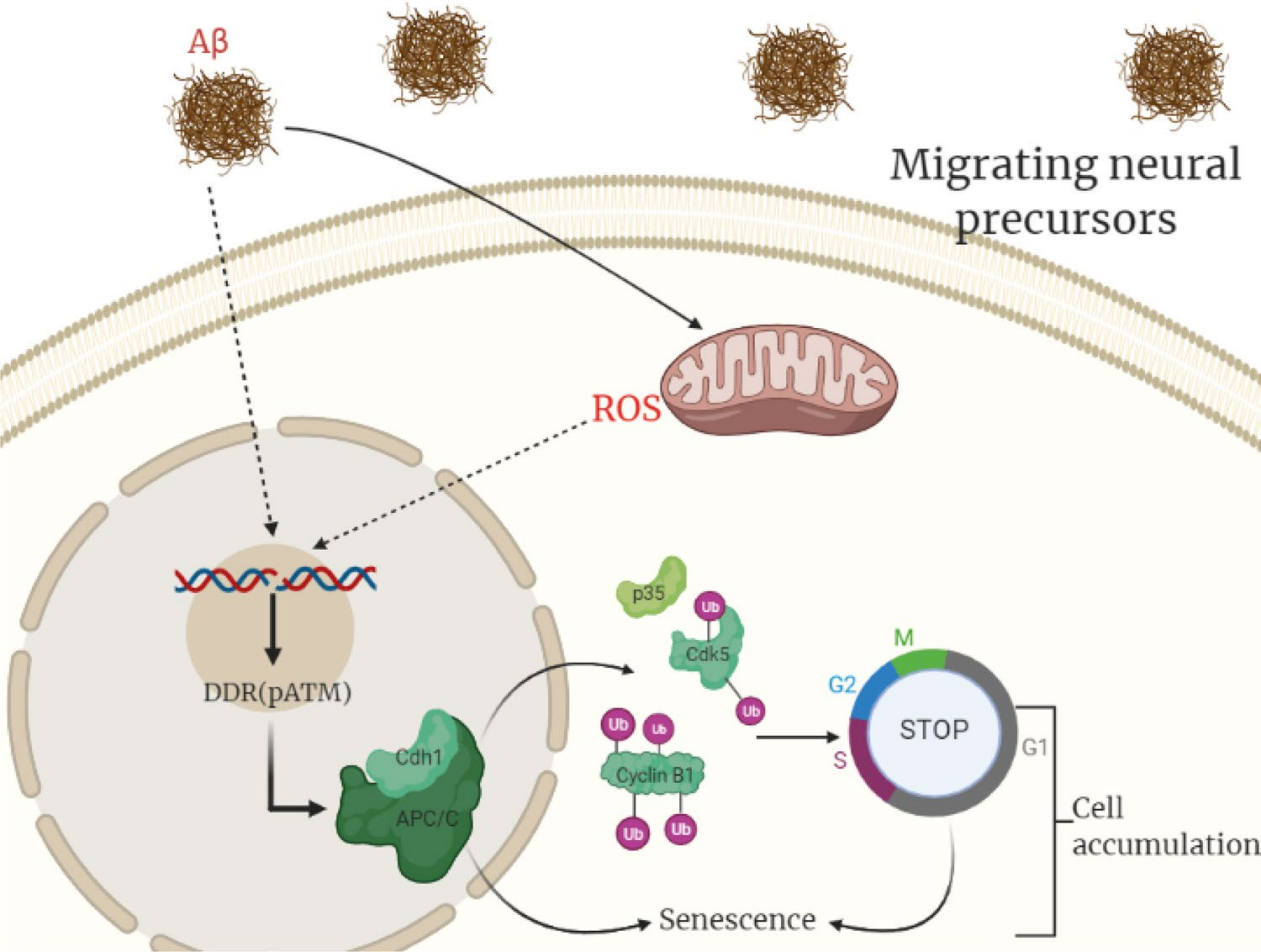
Graphical representation of the proposed molecular pathway.Aβ peptide increases ROS levels in migrating neural precursors (type A cells) leading to DNA damage which provokes a cell cycle arrest associated with an increase of Cdh1 levels and a decrease of its targets cyclin B1 and Cdk5/p35. This cell cycle arrest triggers to a senescence state. Senescent cells accumulate in the V-SVZ and are unable to migrate. Created with BioRender.com

In order to elucidate a plausible molecular explanation for the cell cycle arrest, we studied the master cell cycle regulator APC/C. APC/C is an E3 ubiquitin-ligase that needs as co-factor Cdh1. APC/C-Cdh1 participates in the regulation of G1/S transition and also controls the DNA damage response (DDR) [45, 46]. We observe that Cdh1 levels increase in V-SVZ from APP/PS1 mice indicating that APC/C could be more active [47]. In fact, DDR leads to a degradation of dimethyltransferases of the histone H3K9, mediated by APC/C-Cdh1 activation, and this has been associated with senescence secretory phenotype such as IL-6 secretion [48]. One of the main targets of APC/C-Cdh1 is cyclin B which is polyubiquitinated and degraded to pass to phase S [49–52]. We show that the levels of cyclin B1 decrease in APP/PS1 animals compared to WT, which fits with the observed lower number of cells in phase S. We also show decreased levels of the complex Cdk5/p35 in the transgenic mice, another known target of APC/C-Cdh1 [50] and which is also involved in the neural migration [53]. Figure 5 summarizes our molecular findings.

Interestingly, although we find apoptotic and senescent cells in the V-SVZ of the APP/PS1 mice model, we show that NPCs die mostly by senescence, and on the contrary, astrocytes die mainly by apoptosis. Schneider et al. obtained similar results. They showed that NPCs, following ionizing radiation-induced DNA damage, enter irreversible proliferative arrest with features of cellular senescence and, then, differentiate into astrocytes. This was promoted by ATM [54]. Further, they demonstrated that the differentiated astrocytes enter into apoptosis [55]. Astrocytes have an important role in neural migration so the apoptosis of these cells could also contribute to the migration failure of the migrating neural precursors from the V-SVZ due to its important role in this process [56].

Finally, our results are focused on a new perspective on the role of V-SVZ in neurodegeneration, specifically in AD. The presence of senescence in this neurogenic niche which induces a failure in the migration to the olfactory bulb could fit with the presence of early anosmia seen in AD patients and previously described by many authors, and as we have observed in our mice model [57–59]. In fact, it has been reported that in the adult human brain the V-SVZ/OB via remains active but dimmed compared with other species [60]. Furthermore, the V-SVZ under damage supply astrocytes, oligodendrocytes and new neurons to different parts of the adult brain like the corpus callosum or the striatum, areas seriously affected in the AD pathology [10, 61–64]. The NPC senescence demonstrated to open a new therapeutic target with senolytic compounds such as metformin, rapamycin, aspirin and resveratrol, to improve neurogenesis in the V-SVZ [65–67].

## Data Availability

All data generated or analysed during this study are included in this published article.

## Abbreviations

Aβ: β-Amyloid
AD: Alzheimer’s disease
APC/C: Anaphase-promoting complex
APP: Amyloid precursor protein
ATM: Ataxia telangiectasia mutated
b-Gal: B-galactosidase
Cdh1: CDC20 homologue 1
Cdk5: Cyclin-dependent kinase 5
CDKN2A: Cyclin-dependent kinase inhibitor 2A
CSF: Cerebrospinal fluid
DCX: Doublecortin
EdU: 5-Ethynyl-2′-deoxyuridine
GFAP: Glial fibrillary acid protein
ISO: Isoflurane
NeuN: Neuronal nuclear protein
NGS: Normal goat serum
NPCs: Neural progenitor cells
NSCs: Neural stem cells
PS1: Presenilin 1
PSA-NCAM: Polysialylated-neural cell adhesion molecule
RMS: Rostral migratory stream
SAβGal: Senescence-associated β-galactosidase
SVZ: Subventricular zone
